# Association between *CXCL10* and *DPP4* Gene Polymorphisms and a Complementary Role for Unfavorable *IL28B* Genotype in Prediction of Treatment Response in Thai Patients with Chronic Hepatitis C Virus Infection

**DOI:** 10.1371/journal.pone.0137365

**Published:** 2015-09-04

**Authors:** Kessarin Thanapirom, Sirinporn Suksawatamnuay, Wattana Sukeepaisarnjaroen, Pisit Tangkijvanich, Sombat Treeprasertsuk, Panarat Thaimai, Rujipat Wasitthankasem, Yong Poovorawan, Piyawat Komolmit

**Affiliations:** 1 Division of Gastroenterology, Department of Medicine, Faculty of Medicine, Chulalongkorn University, Bangkok, Thailand; 2 Gastroenterology Unit, Department of Medicine, Srinagarind Hospital, Faculty of Medicine, Khon Kaen University, Khon Kaen, Thailand; 3 Department of Biochemistry, Faculty of Medicine, Chulalongkorn University, Bangkok, Thailand; 4 Center of Excellence in Clinical Virology Department of Pediatrics, Faculty of Medicine, Chulalongkorn University, Bangkok, Thailand; National Taiwan University Hospital, TAIWAN

## Abstract

Pretreatment serum levels of interferon-γ-inducible protein-10 (IP-10, CXCL10) and dipeptidyl peptidase-4 (DPP IV) predict treatment response in chronic hepatitis C (CHC). The association between functional genetic polymorphisms of *CXCL10* and *DPP4* and treatment outcome has not previously been studied. This study aimed to determine the association between genetic variations of *CXCL10* and *DPP4* and the outcome of treatment with pegylated interferon-α (PEG-IFN-α) based therapy in Thai patients with CHC. 602 Thai patients with CHC treated using a PEG-IFN-α based regimen were genotyped for *CXCL10* rs56061981 G>A and *IL28B* rs12979860 C>T. In addition, in patients infected with CHC genotype 1, *DPP4* (rs13015258 A>C, rs17848916 T>C, rs41268649 G>A, and rs 17574 T>C) were genotyped. Correlations between single nucleotide polymorphisms, genotype, and treatment response were analyzed. The rate of sustained virologic response (SVR) was higher for the CC genotype of *IL28B* rs12979860 polymorphisms than for non-CC in both genotype 1 (60.6% vs. 29.4%, *P* < 0.001) and non-genotype 1 (69.4% vs. 49.1%, *P* < 0.05) CHC. SVR was not associated with the *CXCL10* gene variant in all viral genotypes or *DPP4* gene polymorphisms in viral genotype1. Multivariate analysis revealed *IL28B* rs12979860 CC genotype (OR = 3.12; 95% CI, 1.72–5.67; *P* < 0.001), hepatitis C virus RNA < 400,000 IU/ml (OR = 2.21; 95% CI, 1.22–3.99, *P* < 0.05), age < 45 years (OR = 2.03; 95% CI, 1.11–3.68; *P* < 0.05), and liver fibrosis stage 0–1 (OR = 1.64; 95% CI, 1.01–2.65, *P* < 0.05) were independent factors for SVR. Unfavorable *IL28B* rs12979860 CT or TT genotypes with the *CXCL10* rs56061981 non-GG genotype were associated with a higher SVR than GG genotype (66.7% vs. 33.0%, *P* = 0.004) in viral genotype 1. In Thai CHC genotype 1 infected patients with an unfavorable *IL28B* rs12979860 CT/TT genotype, the complementary *CXCL10* polymorphism strongly enhances prediction of treatment response.

## Introduction

Various host genetic factors have been identified as predictors of treatment response in chronic hepatitis C (CHC) infection. From genome-wide association studies, a single nucleotide polymorphism (SNP) near the *IL28B* gene has been found to be strongly associated with treatment response using pegylated interferon α (PEG-IFN-α) and ribavirin therapy [1–3].

The interferon-γ inducible protein of 10 kDa (IP-10), also known as chemokine (C-X-C motif) ligand 10 (CXCL10), is a CXC chemokine and plays a key role in recruitment-activated T lymphocytes and natural killer cells by binding with chemokine receptor 3 (CXCR3). Hepatocytes infected with viral hepatitis C activates the secretion of IP-10 and other chemokines, to stimulate the innate and adaptive immune response [4]. High pretreatment serum IP-10 levels are related to poor treatment outcome for therapy based on PEG-IFN-α in patients with CHC genotype 1 infection [5–7]. Quantification of pretreatment serum IP-10 improves the predictive value of *IL28B* gene polymorphism. Among patients with an unfavorable *IL28B* CT/TT genotype, pretreatment serum concentrations of IP-10 less than 600 pg/ml predicted a sustained virologic response (SVR) [8].

Interestingly, high serum levels of IP-10, which has a potent chemoattractant effect, are associated with treatment failure of PEG-IFN-α and ribavirin. IP-10 is modified by dipeptidyl peptidase IV (DPP IV), which acts by cleaving two amino acid residues from the amino terminus of IP-10 to produce the antagonist form of IP-10 [2], which is capable of binding to CXCR3 (IP-10 receptor) but does not induce signaling [9, 10]. DPP IV is expressed and regulated by T lymphocyte function. High baseline serum soluble DPP IV (sDPP IV) concentration is correlated with poor treatment outcome in patients with CHC genotype 1 infection [11, 12]. Genetic variations in the *CXCL10* and *DPP4* genes alter the expression and binding ability of the IP-10 and DPP IV proteins. A previous study showed that rs56061981, polymorphism of *CXCL10* promoter, modifies the binding affinity of nuclear protein and regulates *CXCL10* expression, which is related to susceptibility to disease progression in chronic hepatitis B infection [13]. The *DPP4* tagging SNPs were significantly associated with *DPP4* methylation, affected mRNA abundance in visceral adipose tissue, and were a cardiovascular risk factor in patients with severe obesity [14, 15].

To our knowledge, there are no data on the association of functional genetic variations of the *CXCL10* and *DPP4* genes and outcome of the treatment with PEG-IFN-α based therapy in CHC infection. This study aimed to demonstrate the prevalence of functional genetic polymorphisms of *CXCL10* rs56061981 G>A, *IL-28B* rs12979860 C>T, *DPP4* rs13015258 A>C, rs17848916 T>C, rs 41268649 G>A, and rs 17574 T>C and its association with treatment outcome in Thai patients with CHC infection treated with PEG-IFN-α and ribavirin. In addition, we investigated the complementary role of *CXCL10* gene polymorphism in patients who had unfavorable *IL28B* genotypes in the prediction of treatment response.

## Materials and Methods

### Study participants

Between June 2012 and November 2013, 602 patients infected with CHC were enrolled, 394 from the Chulalongkorn University Hospital (Bangkok, Thailand), and 208 from the Srinagarind Hospital (Khon Kaen, Thailand). All patients were Thai and fulfilled the following inclusion criteria: a positive test for anti-hepatitis C virus antibody, detectable HCV RNA in serum, treated with standard dose of PEG-IFN-α 2a (180 μg/week) or PEG-IFN-α 2b (1.5 μg/kg/ week) plus ribavirin (800–1400 mg/day). Exclusion criteria were: concomitant human immunodeficiency virus or hepatitis B virus infection, end-stage renal disease, decompensated cirrhosis, post-liver transplantation, and the use of immunosuppressive drugs. Patients’ baseline characteristics, including age, sex, history of alcohol drinking, body mass index, pretreatment HCV RNA, serum alanine aminotransferase, white blood count, hemoglobin, serum sDPP IV concentration, and liver biopsy data were recorded. Patients were classified as having an SVR if HCV RNA was undetectable in the plasma 24 weeks after the completion of therapy. The study protocol was approved by the Institutional Review Board (IRB 562/54) of the Faculty of Medicine, Chulalongkorn University and conducted in compliance with principles of the Declaration of Helsinki. Patients gave written informed consent.

### Characterization of single nucleotide polymorphisms and HCV RNA measurements

Genomic DNA was extracted from peripheral blood leukocytes of 10 ml samples of whole blood using standard phenol-chloroform protocols. Two SNPs, the rs56061981 G>A of the *CXCL10* gene and the rs12979860 C>T of the *IL28B* gene, were genotyped using PCR and restriction fragment length polymorphism analysis in all patients. Four SNPs in the *DPP4* gene (rs13015258 A>C, rs17848916 T>C, rs41268649 G>A, rs17574 T>C) were genotyped in patients with CHC genotype 1.

Human genomic DNA was extracted from 100 μl samples of peripheral blood mononuclear cells incubated with proteinase K in lysis buffer, followed by phenol-chloroform extraction and ethanol precipitation. Finally, the pellet was dissolved in 50 μl sterile water and stored at −20°C until further testing. For amplification, a PCR-specific primer set was designed (primer sequences available on request). Samples of 2 μl of DNA were used to set up 25 μl PCR reactions, using the Perfect *Taq* plus MasterMix (5 PRIME GmbH, Hamburg, Germany) (PCR conditions are available on request). In a total volume of 25 μl, amplified DNA (10 μl) was digested overnight with 2 units of restriction endonuclease using the buffers and temperatures recommended by the manufacturer. *Hinf*I restriction endonuclease was used for *CXCL10* rs56061981, *Brs*I for *DPP4* rs13015258, *Fok*I for *DPP4* rs17848916, and *Dde*I for *DPP4* rs41268649 and *DPP4* rs 17574 (all endonucleases from New England Biolabs, Hitchin, UK). The resulting DNA fragments were determined by 2% agarose gel electrophoresis. The RFLP result became visible under ultraviolet light after staining with ethidium bromide. Quantitative HCV RNA analysis was performed at baseline, weeks 4, 12, 48 during treatment and week 24 after treatment discontinuation using line probe assay INNO-LiPA HCV (Innogenetics, Zwijnaarde, Belgium)

### Serum sDPP IV concentration measurement

Eighty patients (40 patients with SVR and 40 patients without SVR) were randomly selected from the 266 CHC genotype 1 infected patients. From each patient, 6 ml of the peripheral blood sample before treatment was taken into a serum separator tube and allowed to clot for 30 minutes at room temperature before centrifugation for 15 minutes at 1500*g*. The serum was immediately removed and aliquots were stored at −70°C. Human soluble DPP IV (sDPP IV) was quantified using human sDPP IV ELISA (R&D Systems, Minneapolis, MN) and the manufacturer’s protocol, with serum samples diluted 1:100 in sample diluent.

### Statistical analysis

Statistical analysis was performed using SPSS software (version 20.0; IBM, USA). The difference between patients who did and did not achieve SVR was assessed using the chi-squared test for categorical variables and the non-parametric or Student’s *t*-test for continuous variables. Associations of SNPs and responses to treatment were assessed by univariate and multivariate logistic analyses. A value of *P* < 0.05 was considered statistically significant.

## Results

A total of 602 patients with CHC treated with PEG-IFN-α based therapy were enrolled. Of these, 368 patients (61.1%) achieved SVR. All patients were Thai. Baseline patient characteristics are shown in Table 1. There was no difference in most baseline variables between patients with SVR and non-SVR, except that patients without SVR were older and had a higher proportion of HCV genotype 1 infection, baseline HCV RNA, and advanced liver fibrosis than patients with SVR.

**Table 1:** Baseline characteristic of patients with CHC infection.

Baseline characteristics	Non-SVR (n = 234)	SVR (n = 368)	p-value
**Female, n (%)**	77 (32.9%)	128 (34.8%)	0.64
**Age < 45 years, n (%)**	36 (15.5%)	99 (27.2%)	0.001
**Body mass index (kg/m** ^**2**^ **), mean ± SD**	24.6 ± 3.4	24.5 ± 3.5	0.77
**Alcohol drinking, n (%)**	135 (69.2%)	152 (61.3%)	0.08
**Diabetes Mellitus, n (%)**	49 (24.7%)	54 (21.4%)	0.40
**Genotype**			
**1**	126 (54.3%)	140 (39.1%)	< 0.05
**2**	0 (0.0%)	1 (0.3%)	
**3**	92 (39.7%)	174 (48.6%)	
**6**	14 (6.0%)	43 (12.0%)	
**Pre-treatment HCV-RNA ≥ 400,000 IU/mL, n (%)**	188 (85.1%)	222 (67.9%)	0.000
**Pre-treatment ALT level (U/L), mean ± SD**	105 ± 162	102 ± 76	0.76
**Pre-treatment AST level (U/L), mean ± SD**	87 ± 133	73 ± 51	0.07
**Pre-treatment ALP level (U/L), mean ± SD**	93 ± 39	90 ± 53	0.55
**Advanced fibrosis (stage 2–4), n (%)**	71 (50.4%)	73 (36.1%)	0.009
**PEG-IFN-α 2a, n (%)**	108 (55.7%)	152 (57.8%)	0.16
**PEG-IFN-α 2b, n (%)**	82 (42.3%)	97 (36.9%)	0.37

Abbreviation: AST, aspartate aminotransferase; ALT, alanine aminotransferase; ALP, alkaline phosphatase.

### Prevalence of *CXCL10* rs56061981 and *IL28B* rs12979860 in Thai patients with chronic hepatitis C

The genotypic distribution of *CXCL10* rs56061981 and *IL28B* rs12979860 was shown in Table 2. Most patients with CHC infection in this study had the GG allele of *CXCL10* rs56061981 (77.2%) and favorable *IL28B* CC allele (80.4%).

**Table 2:** Genotypic frequencies of *CXCL10* rs56061981 and *IL28B* rs12979860 in Thai patients with CHC infection.

	All patients (n = 602)	Genotype 1 (n = 266)	Non-genotype 1 (n = 336)
***CXCL10* rs 56061981**			
**GG**	465 (77.2%)	199 (74.8%)	267 (79.4%)
**GA**	132 (21.9%)	66 (24.8%)	65 (19.4%)
**AA**	5 (0.8%)	1 (0.4%)	4 (1.2%)
***IL28B* rs12979860**			
**CC**	484 (80.4%)	198 (74.6%)	281 (83.7%)
**CT**	108 (18.0%)	61 (22.8%)	51 (15.1%)
**TT**	10 (1.6%)	7 (2.6%)	4 (1.2%)

### Association of *CXCL10* rs56061981 and *IL28B* rs12979860 polymorphisms and treatment outcome using PEG-IFN-α based therapy in Thai patients with chronic hepatitis C

**Fig 1:**
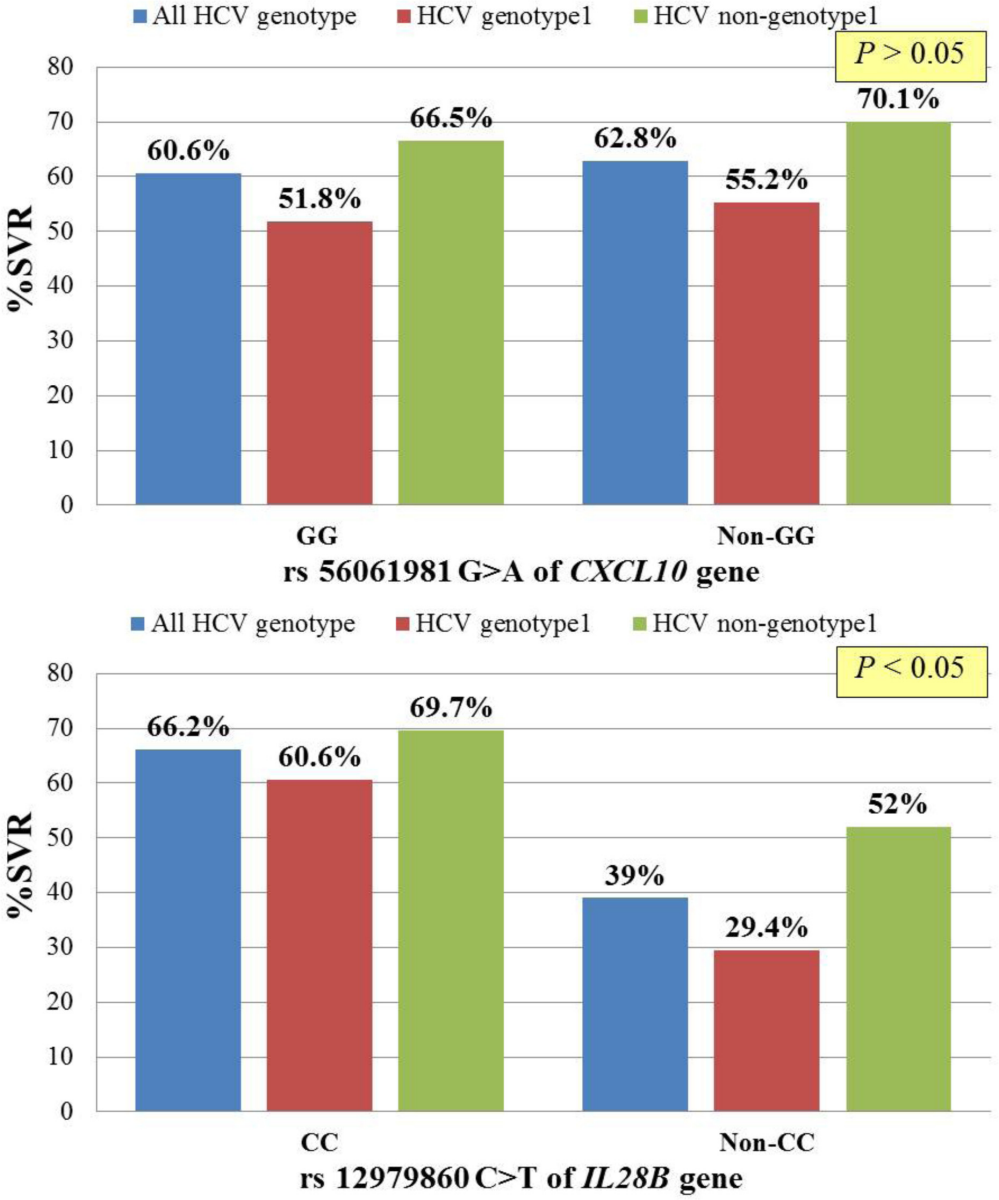
*CXCL10* rs56061981, *IL28B* rs12979860 polymorphisms according to sustained virological response (SVR) in patients with CHC infection.

The genotypic distribution of the *CXCL10* and *IL28B* variants and treatment response classified by HCV genotype are summarized in Fig 1. There was no association between rs56061981 promoter polymorphism of *CXCL10* and SVR in patients with CHC genotype1 and non-genotype 1 infection (*P* > 0.05). By contrast, SNP at rs12979860 in *IL28B* predicted SVR in patients with both CHC genotype 1 (*P* < 0.001) and non-genotype 1 infection (*P* < 0.05).

In the univariate analysis across all HCV genotypes, age, HCV genotype, baseline HCV RNA, liver fibrosis, and *IL28B* rs12979860 polymorphism was linked to treatment response. Stepwise logistic regression analysis was used to assess independent predictors for good treatment outcomes. Multivariate analysis revealed that *IL28B* rs12979860 CC genotype (OR = 3.12; 95% CI, 1.72–5.67; *P* < 0.001), HCV RNA < 400,000 IU/ml (OR = 2.21; 95% CI, 1.22–3.99; *P* < 0.05), age < 45 years (OR = 2.03; 95% CI, 1.11–3.68; *P* < 0.05) and liver fibrosis stage 0–1 (OR = 1.64; 95% CI, 1.01–2.65; *P* < 0.05) were independent factors for SVR. During treatment, 66.7% and 88.4% of patients had undetectable HCV RNA at week 4 (rapid virologic response or RVR) and week 12 (early virologic response or EVR), respectively. The rate of SVR was higher in patients who achieved RVR (72.3% vs. 27.7%; OR = 4.62, 95% CI, 3.07–6.98; *P* < 0.001) and EVR (OR = 10.74, 95% CI = 5.14–22.46, *P* < 0.001) than patients without RVR and EVR, respectively.

### Combining assessment of *CXCL10* rs56061981and *IL28B* rs12979860 polymorphisms

**Fig 2:**
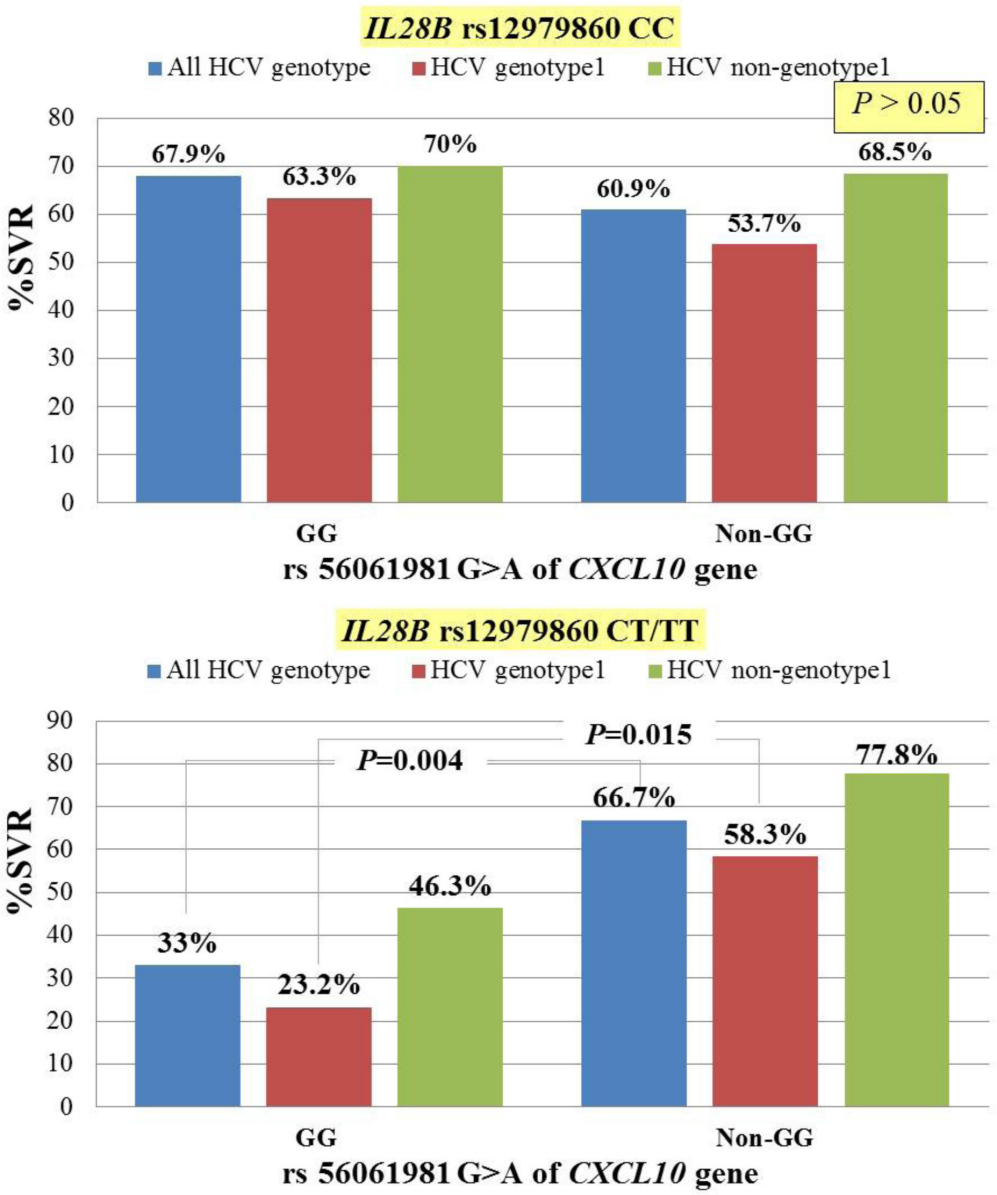
Association of *CXCL10* rs56061981 polymorphism and treatment response in CHC-infected patients who had *IL28B* CT/TT and CC genotypes.

Although the relationship between *IL28B* CT or TT genotypes and poor treatment outcome is well known, 15–30% of patients in this group achieved SVR [1, 16]. A previous study found that combining *IL28B* genotype and baseline IP-10 level improves the predictive value for treatment response [8]. This study demonstrated that use of the functional promoter polymorphism *CXCL10* rs56061981 had an important role in predicting SVR in patients infected with CHC who had unfavorable *IL28B* rs12979860 CT or TT genotypes. In our study, 118 patients (19.6%) had *IL28B* rs12979860 CT or TT genotypes. Of these, 57.6% (n = 68) had HCV genotype1 infection. Correlation of *CXCL10* rs56061981 and treatment response across total, genotype 1 and non-genotype 1 HCV infected patients who had favorable and unfavorable *IL28B* rs12979860 was shown in Fig 2. The *CXCL10* rs56061981 non-GG genotype was associated with a higher rate of SVR achievement than GG genotype only in patients with HCV genotype 1 infection that had unfavorable *IL28B* rs12979860. By univariate analysis, HCV RNA < 400,000 IU/ml and *CXCL10* rs56061981 polymorphism was related with SVR. Multivariate analysis showed in this group of patients, baseline *CXCL10* rs56061981 non-GG genotype (OR = 4.00, 95% CI, 1.03–15.53; *P* = 0.045) strongly predicted SVR.

### Serum sDPP IV concentration, *DPP4* rs13015258 A>C, *DPP4* rs17848916 T>G, *DPP4* rs41268649 G>A, and *DPP4* rs17574 T>C variants and treatment outcome in hepatitis C virus genotype 1

**Fig 3:**
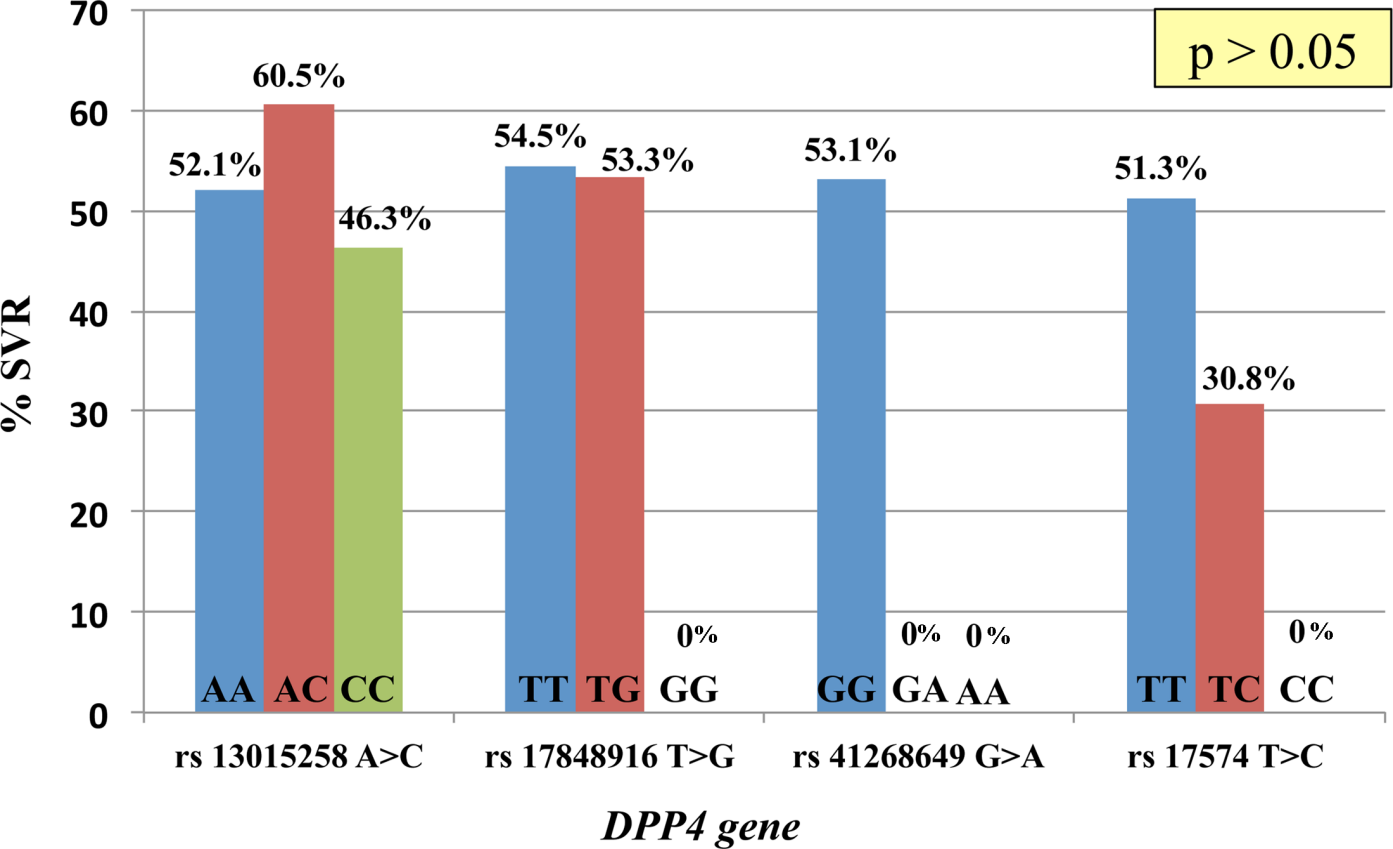
*DPP4* rs13015258 A>C, *DPP4* rs17848916 T>G, *DPP4* rs41268649 G>A, *DPP4* rs17574 T>C genotype and treatment outcome in Thai patients with CHC genotype1 infection.

The prevalence of four SNPs in *DPP4* gene of patients with CHC genotype 1 is shown in Fig 3. The SVR rate is not associated with *DPP4* gene polymorphisms. Eighty patients with genotype 1 infection treated with PEG-IFN-α and ribavirin (40 patients with SVR and 40 patients without SVR) were evaluated for sDPP IV concentration. Baseline characteristics of these selected 80 patients compared with the remaining patients were shown in S1 Table. The selected patients had more advanced age and less history of alcohol drinking. The mean and standard deviation of sDPP IV were not different between patients who did or did not have SVR (695 ± 191 ng/ml vs. 697 ± 170 ng/ml, *P* = 0.67).

### Serum sDPP IV and *DPP4* rs13015258 A>C, *DPP4* rs17848916 T>G, *DPP4* rs41268649 G>A, and *DPP4* rs17574 T>C polymorphisms

**Fig 4:**
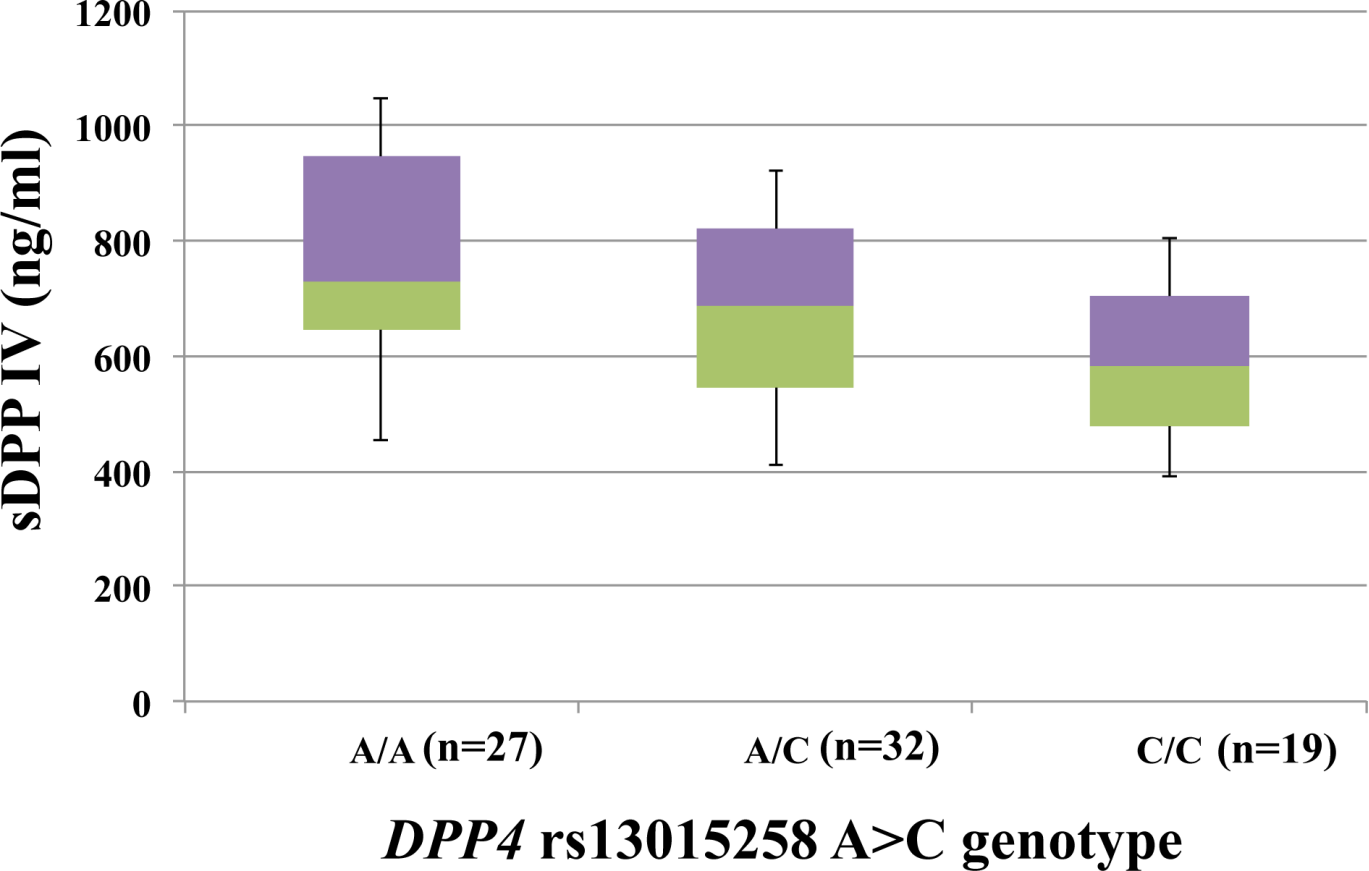
Correlation of soluble DPP IV and polymorphism of *DPP4* rs13015258 A>C in patients with chronic hepatitis C genotype 1 infection treated with PEG-IFN-α and ribavirin. Statistics analysis using two-tailed Mann-Whitney U-test.

The correlation between four SNPs of *DPP4* and sDPP IV concentration was assessed. There was a trend of increasing mean sDPP IV in patients with *DPP4* rs 13015258 AA, AC, and CC genotypes (749, 703, and 585 ng/ml), respectively, as shown in Fig 4. Serum sDPP IV concentration were significantly higher in patients with *DPP4* rs13015258 AA than in patients with the CC genotype, *P* = 0.006. Mean sDPP IV concentration in patients with *DPP4* rs17848916 TT vs. TG genotype (694 vs. 632 ng/ml, *P* = 0.44) or *DPP4* rs 17574 TT vs. TC genotype (709 vs. 634 ng/ml, *P* = 0.26) were not significantly different. All patients had *DPP4* rs41268649 GG genotype; mean sDPP IV was 690 ng/ml.

## Discussion

To our knowledge, this is the first study to attempt to demonstrate the association between functional genetic variation of *CXCL10*, *IL28B* and *DPP4* and treatment outcome with PEG-IFN-α and ribavirin in Thai patients with CHC infection.

The main results of the present study are:

- In Thai patients infected with CHCs genotype 1 who had unfavorable *IL28B* rs12979860 CT or TT genotypes, using the *CXCL10* rs5606198 polymorphism improved the prediction of SVR with PEG-IFN-α based therapy.
- The *IL28B* rs12979860 CC genotype was a good predictor for SVR among Thai patients infected with CHC.
- There was a high prevalence of *IL28B* rs12979860 CC genotype in Thai patients with CHC infection.

IP-10 is an essential chemokine, stimulating the immune response against HCV infection. A high pretreatment serum IP-10 concentration predicts treatment failure in patients with CHC infection treated with PEG-IFN-α and ribavirin therapy [5–7, 17, 18]. In acute HCV infection, high baseline levels of IP-10 are associated with failure of spontaneous clearance of HCV; patients with high baseline levels of IP-10 should therefore be considered for early therapeutic intervention [19, 20]. Serum levels and hepatic expression of IP-10 correlate with severity of liver histopathology in patients with CHC infection [21, 22]. DPP IV affects the immune system through HCV-specific T-cell activation and various chemokine modifications, including IP-10 [9, 23, 24]. Patients with HCV infection show increased serum DPP IV expression [25, 26]. High baseline sDPP IV is related to poor treatment response [11]. Previous studies have showed that functional SNPs of *CXCL10* and *DPP4* affect their function. However, this study demonstrated that SNPs rs56061981 in *CXCL10* and rs13015258, rs17848916, rs 41268649, and rs17574 in *DPP4* were not associated with treatment response in CHC-infected Thai patients treated with PEG-IFN-α based therapy. The reason for this could not be clearly identified. Possible mechanisms could arise during the process after transcription and translation or other chemokine-complex-modifying effects of IP-10 and DPP IV. The present study did not evaluate serum IP-10 because a fraction of the patients had been enrolled during or after therapy. This prevented us from evaluating pretreatment IP-10 levels and analyzing possible effects of the studied polymorphisms on serum IP-10 level. However, functional analysis from Deng G et al showed that *CXCL10* rs56061981 polymorphism alters the binding affinity of nuclear protein and regulates *CXCL10* expression, but does not correlate with IP-10 level [13]. In contrast, Mihm S et al found that serum IP-10 is strongly associated with *CXCL10* gene expression in CHC infection [27]. We are interested in demonstrating the relationship of treatment response and the variation of host genetic factor. In healthy Chinese population, the *CXCL10* rs56061981 genotypic frequencies were 86.2%, 12.7% and 1.1% for GG, GA and AA genotypes, respectively [28]. This current study found that the *CXCL10* rs56061981 non-GG genotype was frequent in Thai patients with CHC compared to the healthy Chinese population. Data of the studied *DPP4* polymorphisms of general population have not been reported.

The *IL28B* rs12979860 polymorphism on chromosome 19, which encodes IFN-λ, is associated with spontaneous HCV clearance and treatment response to PEG-IFN-α and ribavirin [1, 2, 29]. The highly variable prevalence of the favorable *IL28B* genotype in patients of varying ethnicity, with Africans having the lowest frequencies and East Asians the highest, may explain the difference in SVR among different populations. All patients in this study were Thai, and there was a high prevalence of the favorable *IL28B* CC genotype (79.9%). Serum levels of IP-10 and sDPP IV improve the predictive value of *IL28* variants for treatment response. Low baseline concentrations of serum IP-10 and sDPP IV improved the likelihood of achieving SVR among patients infected with CHC genotype 1 who had unfavorable *IL28B* rs12979860 CT and TT genotypes [8, 11]. Interestingly, this study demonstrated that genetic variant rs56061981 non-GG genotypes in the promoter region of *CXCL10* is related to SVR in patients who have *IL28B* rs12979860 CT or TT genotypes and might contribute to a genome-based personalization therapy, even in this era of direct-acting antivirals. This study indicates that not only is serum IP-10 level a prognostic factor for SVR, but that including analysis of *CXCL10* rs56061981 also improves prediction treatment response for unfavorable *IL28B* variants with high specificity in Thai patients with CHC genotype 1.

Despite moving to the new era of CHC treatment with direct-acting antiviral agents (DAA), in most developing countries, including Thailand, where DAAs are not widely accessible, the PEG-IFN-α based treatment remains an effective therapy for CHC infection. Several factors are potential predictors for treatment response with PEG-IFN-α based regimen and may improve predictive efficiency of *IL-28B* genotype for treatment outcome in CHC-infected patients, including baseline γ-glutamyltransferase/alanine aminotransferase ratio, vitamin D level, serum ferritin concentration, and early anemia. However, available data are contradictory [30–35]. In this current study, we did not collect baseline γ-glutamyltransferase, serum vitamin D and ferritin. Additionally, we intended to study the association of pre-treatment factors and SVR, thus we did not include RVR or EVR in multivariate analysis for SVR. However, we found in univariate analysis that RVR and EVR during therapy predicted SVR.

Our study is limited in that sDPP IV concentration and *DPP4* genotyping was performed only for limited number of patients with HCV genotype 1 infection, since previous data had found that the association with SVR was found in HCV genotype 1, and this was a secondary objective. Further studies are warranted to assess the relation between sDPP IV concentration, *DPP4* gene variants and treatment outcome in patients with CHC infection.

## Supporting Information

S1 Table Baseline characteristics of CHC genotype 1 infected patients with and without pre-treatment sDPP IV. (PDF)
