# Supplementary material for: Association between CXCL10 and DPP4 Gene Polymorphisms and a Complementary Role for Unfavorable IL28B Genotype in Prediction of Treatment Response in Thai Patients with Chronic Hepatitis C Virus Infection
Source: PLoS One. 2015 Sep 4;10(9):e0137365. doi: 10.1371/journal.pone.0137365 (PMC4560372; doi:10.1371/journal.pone.0137365)
Supplement: S1 Table — (PDF) [file pone.0137365.s001.pdf]

S1 Table: Baseline characteristics of CHC genotype 1 infected patients with and without pre-treatment sDPP IV.

Baseline characteristics	No pre-treatment sDPP IV evaluation (n=186)	Availability of pre-treatment sDPP IV evaluation (n=80)	p-value
**Female, n (%)**	46 (24.7)	37 (46.2)	< 0.05
**Age < 45 years, n (%)**	30 (16.3)	15 (19.0)	0.60
**Body mass index (kg/m^2), mean ± SD**	24.6 ± 3.4	24.8 ± 3.5	0.38
**Alcohol drinking, n (%)**	117 (85.4)	35 (44.3)	< 0.001
**Diabetes Mellitus, n (%)**	32 (22.9)	22 (27.8)	0.41
**Pre-treatment HCV-RNA ≥ 400,000 IU/mL, n (%)**	144 (82.3)	61 (82.4)	0.98
**Pre-treatment ALT level (U/L), mean ± SD**	114.2 ± 183.9	85.4 ± 45.3	0.98
**Pre-treatment AST level (U/L), mean ± SD**	85.1 ± 146.5	65.9 ± 34.3	0.99
**Pre-treatment ALP level (U/L), mean ± SD**	92.1 ± 36.7	82.3 ± 29.2	0.14
**Advanced fibrosis (stage 2-4), n (%)**	50 (41.7)	14 (38.9)	0.77
**PEG-IFN-α 2a, n (%)**	71 (50.0)	44 (62.0)	0.10
